# Differential Regulation of Stomatal Conductance as a Strategy to Cope With Ammonium Fertilizer Under Ambient Versus Elevated CO_2_

**DOI:** 10.3389/fpls.2019.00597

**Published:** 2019-05-24

**Authors:** Fernando Torralbo, María Begoña González-Moro, Edurne Baroja-Fernández, Iker Aranjuelo, Carmen González-Murua

**Affiliations:** ^1^ Department of Plant Biology and Ecology, University of the Basque Country (UPV/EHU), Bilbao, Spain; ^2^ Instituto de Agrobiotecnología (IdAB)-CSIC, Mutilva, Spain

**Keywords:** ammonium, nitrate, wheat, elevated CO_2_, N assimilation, stomata conductance, aquaporins

## Abstract

While nitrogen (N) derived from ammonium would be energetically less expensive than nitrate-derived N, the use of ammonium-based fertilizer is limited by the potential for toxicity symptoms. Nevertheless, previous studies have shown that exposure to elevated CO_2_ favors ammonium assimilation in plants. However, little is known about the impact of different forms of N fertilizer on stomatal opening and their consequent effects on CO_2_ and H_2_O diffusion in wheat plants exposed to ambient and elevated CO_2_. In this article, we have examined the response of the photosynthetic machinery of durum wheat (*Triticum durum*, var. Amilcar) grown with different types of N fertilizer (NO_3_^−^, NH_4_^+^, and NH_4_NO_3_) at 400 versus 700 ppm of CO_2_. Alongside gas exchange and photochemical parameters, the expression of genes involved in CO_2_ (*PIP1.1* and *PIP2.3*) and H_2_O (*TIP1*) diffusion as well as key C and N primary metabolism enzymes and metabolites were studied. Our results show that at 400 ppm CO_2_, wheat plants fertilized with ammonium as the N source had stress symptoms and a strong reduction in stomatal conductance, which negatively affected photosynthetic rates. The higher levels of *PIP1.1* and *PIP2.3* expression in ammonium-fertilized plants at 400 ppm CO_2_ might reflect the need to overcome limitations to the CO_2_ supply to chloroplasts due to restrictions in stomatal conductance. This stomatal limitation might be associated with a strategy to reduce ammonium transport toward leaves. On the other hand, ammonium-fertilized plants at elevated CO_2_ did not show stress symptoms, and no differences were detected in stomatal opening or water use efficiency (WUE). Moreover, similar gene expression of the aquaporins *TIP1*, *PIP1.1,* and *PIP2.3* in ammonium-fertilized plants grown at 700 ppm compared to nitrate and ammonium nitrate plants would suggest that an adjustment in CO_2_ and H_2_O diffusion is not required. Therefore, in the absence of a stress context triggered by elevated CO_2_, ammonium- and ammonium nitrate-fertilized plants were able to increase their photosynthetic rates, which were translated eventually into higher leaf protein content.

## Introduction

The increase in the world’s population predicted for the end of the twenty-first century^1^ will require that intensive agriculture employs large amounts of nitrogen (N) to increase maximum cereal yields.

Both nitrate and ammonium are the major N forms available in soils for plants (Andrews et al., 2013; Tegeder and Masclaux-Daubresse, 2017). In both natural and agricultural soils, microbial processes in soils favor a mixed nutrition with fluctuating contents of the respective sources depending on the environmental conditions of temperature and soil water content and the type of fertilizers applied, among other factors (Benckiser et al., 2015). In addition, when both inorganic-N sources are available in plants, ammonium is preferentially taken up and assimilated into organic molecules due to its more chemically reduced status than nitrate, thus saving metabolic energy during its assimilation (Andrews et al., 2013). Nevertheless, the application of ammonium as the sole N source generally elicits toxicity symptoms in many plant species (Britto and Kronzucker, 2002). In addition to the visual symptoms expressed as a global reduced plant growth or leaf chlorosis, excess ammonium causes an alteration in expression and activity of N-assimilating enzymes, disruptions in photosynthesis and hormonal homeostasis, deficiency in ion balance, or induction of higher photorespiration rates (Guo et al., 2007; Esteban et al., 2016). However, these effects are dependent on plant species, variety, or accession, as well as external ammonium availability (Britto and Kronzucker, 2002; Esteban et al., 2016). In addition, environmental factors such as light intensity (Ariz et al., 2011; Setién et al., 2013), pH conditions (Sarasketa et al., 2016), the external N concentration (Vega-Mas et al., 2015), and atmospheric CO_2_ concentration (Nimesha et al., 2017; Rubio-Asensio and Bloom, 2017; Vega-Mas et al., 2017) determine the threshold for ammonium toxicity. According to these studies, an adequate availability of photoassimilates is essential to maintain the internal ammonium homeostasis in the plant cell, thus avoiding toxic effects associated with ammonium nutrition (Vega-Mas et al., 2017).

Plants modulate stomatal opening in order to optimize the rate of CO_2_ diffusion and to minimize water loss, having a key role in photosynthetic machinery and water transport. The regulation of stomatal opening and closure has an important effect in limiting transpiration and therefore on water and nutrient transport through the plant. Increasing CO_2_ concentrations, exposure to heavy metals, and saline and soil water stress are environmental stimuli described as reducing the transpiration rate (Long et al., 2004; Bastías et al., 2010; Jauregui et al., 2015; Rucińska-Sobkowiak, 2016). Although exposure to elevated CO_2_ and toxic compounds such as surplus external ammonium induces stomatal closure (López-Millán et al., 2009; Aranjuelo et al., 2014), information on the involvement of ammonium in water transport within the plant is scarce. In addition to stomatal regulation, most water transport is mediated by aquaporins (AQPs) (Maurel et al., 1997; Henzler et al., 2004). Moreover, these water transport proteins have also been reported to have a role in responding to salinity, osmotic stress, or ammonia transport (Forrest and Bhave, 2007). More specifically, to maintain water balance and photosynthetic performance because AQPs can also mediate the flux of small non-charged molecules, in particular CO_2_, ammonium/ammonia, and urea (Ding et al., 2018; Pawłowicz and Masajada, 2019). Therefore, stomatal regulation together with the regulation of AQPs (abundance or activity) in plants may be essential to withstand future scenarios of elevated CO_2_ conditions and changing agricultural N management.

Plants are able to adapt their biochemical and physiological strategies to assimilate different N sources into organic compounds under changing CO_2_ atmospheres. The predicted rise in atmospheric CO_2_ would favor plant development under a nutrition based on ammonium fertilization because larger quantities of photoassimilates would be available for primary ammonium assimilation (Vega-Mas et al., 2015). Indeed, an elevated CO_2_ atmosphere promotes plant growth, which in turn overcomes the associated ammonium toxicity (Vega-Mas et al., 2015). Nevertheless, the photosynthetic response to elevated CO_2_ does not only depend on CO_2_ as a substrate, but also other physiological and environmental factors have effect on that response (Xu et al., 2016). Frequently, under elevated CO_2_ conditions, plants decrease stomatal conductance, which avoids water loss and increases water use efficiency (WUE). Under this scenario, the modulation of AQPs may be a determinant for absorption and assimilation of different N sources.

The present work has been conceived to evaluate the effect of elevated CO_2_ conditions on stomatal functioning and aquaporins involved in the uptake of water and nutrients in wheat plants. For this, durum wheat plants (*Triticum durum* Def. cv. Amilcar) were grown at two different concentrations of atmospheric CO_2_ (400 and 700 ppm) on different forms of N nutrition (NO_3_^−^, NH_4_^+^, and NH_4_NO_3_) for 2 weeks after a nitrate adaptation over 5 weeks. We observed that stomatal conductance is central to the responsiveness of ammonium-fertilized plants. Stomatal closure acts under ambient CO_2_ conditions to prevent massive ammonium accumulation in shoots, while at elevated CO_2_ conditions, the stomatal aperture ameliorates carbon and ammonium assimilation and minimizes symptoms of stress symptoms derived from ammonium nutrition.

## Materials and Methods

### Plant Material and Experimental Design

Seeds of wheat plants (*Triticum durum* L. cv. Amilcar) were germinated on trays filled with perlite:vermiculite (1:1, v:v) and watered with deionized water. Seeds were maintained for 10 days in darkness and at 4°C to synchronize the germination. After this, seedlings were transferred to 5 L hydroponic pots in two independently controlled environmental chambers (Phytotron Service, SGIker, UPV/EHU), under 550 μmol m^−2^ s^−1^ light intensity, 25/17°C temperature, and 50/60% relative humidity during the 14/10 h of the day/night-photoperiod, respectively. Plants were grown under two different controlled atmospheres of 400 and 700 ppm CO_2_ levels. Hoagland solution (Arnon and Hoagland, 1940) was replaced three times per week. Wheat plants at Z39 (Zadoks scale) were grown for 5 weeks under nitric nutrition based on calcium nitrate. Afterward, for the following 2 weeks, the N source was modified with ammonium sulfate (NH_4_^+^) or ammonium nitrate (NH_4_NO_3_), keeping in parallel a set of control plants under nitrate nutrition (NO_3_^−^). The N source was supplied at a rate of 10 mM N. Gas exchange determinations were measured in flag leaves of wheat plants at Z51 (Zadoks scale) between 2 and 5 h after onset the photoperiod. After gas exchange parameter determination in flag leaves of four plants from each of the CO_2_ condition, shoot of wheat plants was harvested and dried in an oven at 80°C for 72 h for shoot biomass determination. For metabolic and enzymatic analysis, totally expanded flag leaves of four plants were harvested and stored at −80°C until further measurements.

### Gas-Exchange Determinations

Gas-exchange measurements were conducted in totally expanded flag leaves using a Li-COR 6400XP portable photosynthesis system (LI-COR Inc., Lincoln, NE, USA). The rate of CO_2_ assimilation (A_N_), stomatal conductance (g_s_), and intercellular CO_2_ (Ci) parameters was determined under light-saturated conditions with a photosynthetic photon flux density (PPFD) of 1,200 μmol m^−2^ s^−1^ at 25°C and with the reference CO_2_ concentration of the respective growth chamber. The instantaneous water use efficiency (WUEi) was determined dividing the rate of CO_2_ assimilation by the stomatal conductance (A_N_/g_s_). The thylakoid electron transport rate (ETR) and maximal quantum efficiency of PSII (*F*_v_/*F*_m_) were measured using a Leaf Chamber Fluorometer (LFC 6400–40; Li-COR) coupled to the Li-COR 6400XT portable photosynthesis system 45 min after the dark period started.

### Metabolite Determination

Starch was measured from 10 mg of lyophilized powdered samples of flag leaves by modified hydroalcoholic extraction as described by Fuertes-Mendizábal et al. (2010). The dry residue obtained in the hydroalcoholic extraction was resuspended, and starch was determined as glucose equivalents by using a test kit (Boehringer Mannheim, Germany) after α-amylase and amyloglucosidase digestion. Maltose was determined as described by Baslam et al. (2017). Briefly, 0.1 g of frozen plant powder was resuspended in 1 ml of 90% ethanol, incubated for 90 min at 70°C, and centrifuged at 13,000 *g* for 10 min. Maltose from supernatants was then determined by HPAEC-PAD on a DX-500 Dionex system by gradient separation with a CarboPac PA20 column according to the application method suggested by the supplier. Then, the extract was centrifuged at 13,000 *g* for 10 min. Inorganic forms of N as nitrate and ammonium were determined according to the methods described by Patton and Crouch (1977) and Cataldo et al. (1974), respectively.

The free amino acid profile was quantified at the Scientific and Technological Center of the University of Barcelona (CCiT UB). Amino acids were extracted from flag leaves homogenized with 1 M HCl (1:20, w:v). After 16 h of incubation at −20°C, the extracts were centrifuged at 10,000 *g* for 15 min and filtered. Norleucine was added as an internal standard to the five-time diluted amino acid extraction. Afterward, 20 μl of derivatized sample was injected for amino acid determination by HPLC using Waters Delta 600 chromatographic system with a column (Nova-Pak C18 4 μm, 3.9 × 150 mm) and an absorbance detector (Waters 2,487 Dual λ) coupled to an auto sampler (Waters 717plus) using the AccQTag pre-column derivatization method. The reaction of amino acids with 6-aminoquinolyl-N-hydroxysuccinimidyl carbamate yields derivatives that are detected at 254 nm, and their concentrations were calculated according to internal standards (Cohen and Michaud, 1993; Cohen and De Antonis, 1994).

### C Cycle Enzymatic Activities

Powdered frozen flag leaves were homogenized in an extraction buffer consisting of 100 mM HEPES pH 7.5, 2 mM EDTA, 2 mM dithiothreitol, 1 mM PMSF, 10 μl ml^−1^ protease inhibitor cocktail (Sigma P9599), and centrifuged at 14,000 *g* for 20 min. The supernatant was desalted by ultrafiltration on Vivaspin 500 centrifugal concentrator (Sartorius), and the protein extract thus obtained was assayed for enzymatic activities. Both α- and β-amylolytic activities were assayed as described by Sweetlove et al. (1996). Soluble starch synthase activity was measured in two steps: (1) incubation in a buffer reaction with 50 mM HEPES pH 7.5, 6 mM MgCl_2_, 3 mM dithiothreitol, 1 mM adenosine diphosphate glucose (ADPG), and 3% glycogen for 5 min at 37°C. After stopping the reaction by boiling for 2 min, (2) the ADP produced was measured by HPLC on a Waters Associates system fitted with a Partisil-10-SAX column. One unit (U) is defined as the amount of enzyme that catalyzes the production of 1 μmol of product per min.

### N Cycle Enzymatic Activities

Soluble protein was extracted from powdered frozen flag leaves homogenized with extraction buffer (1:20, w:v) based on Gibon et al. (2004). Soluble protein was measured according to Bradford (1976) from the supernatant recovered after centrifugation at 4,000 *g* for 30 min at 4°C. Nitrate reductase (NR) was determined as the maximum activity by incubating 50 μl of protein extract for 30 min at 30°C according to Baki et al. (2000). Glutamine synthetase (GS) and aminating-glutamate dehydrogenase activity (NADH-GDH) were determined as described by Sarasketa et al. (2016).

### RNA Extraction and Quantitative Real-Time PCR

Total RNA was isolated from pulverized flag leaves using a Nucleospin RNA plant kit (Macherey-Nagel) according to the manufacturer’s recommendations. RNA integrity and purity were checked on a 1.5% (v:v) agarose gel, and 1 μg of RNA was retrotranscribed into cDNA using the PrimeScript™ RT reagent kit (Takara Bio Inc.). Gene expression was determined using a StepOne Plus Real-Time PCR System (Applied Biosystems) in a 15 μl reaction using the SYBR Premix ExTaq™ (Takara Bio Inc.), 200 nM of each gene-specific primer of the N-transporter (Vicente et al., 2015) and aquaporin (Jauregui et al., 2015) genes, and 2 μl of cDNA diluted 1:10. The PCR thermal profile was: 95°C for 10 min, 40 cycles (95°C for 15 s and 60°C for 1 min) and a final step to obtain the melting curve. Relative expression was calculated as the ∆Cp between each gene and the geometric average of the reference genes actin and RNase L inhibitor-like protein (Giménez et al., 2011).

### Statistical Analysis

Data sets were analyzed using IBM SPSS Statistics 22 software (Armonk, NY, USA). Normality of residuals and homogeneity of variance were analyzed with the Kolmogorov-Smirnov and Levene tests. The data that did not pass normality test were transformed. The effect size of each factor and their interactions were determined with the use of partial eta-squared, which describe a proportion of variability in a sample associated with an independent variable. Significant differences between N treatments and CO_2_ conditions were analyzed using multifactorial ANOVA followed by LSD test. All analyses were performed at a significance level, *p* < 0.05.

## Results

Growth of the durum wheat cv. Amilcar showed different behaviors depending on the N form supplied in the nutrient solution and the CO_2_ environmental conditions. Both nitrate and ammonium nitrate nutrition showed the highest biomass production, regardless the CO_2_ conditions. However, plants grown under exclusive ammonium nutrition showed a biomass 32 and 42% lower than nitrate-fertilized plants at ambient and elevated CO_2_ conditions, respectively (Table 2). In addition, plants fertilized with mixed nutrition of ammonium nitrate had increased shoot biomass compared to the respective plants at ambient CO_2_. However, no CO_2_-effect was observed for ammonium nutrition; thus, the biomass of plants grown under ammonium nutrition was reduced by about 40% with respect to the treatment fertilized with nitrate (Table 2).

**Figure 1:**
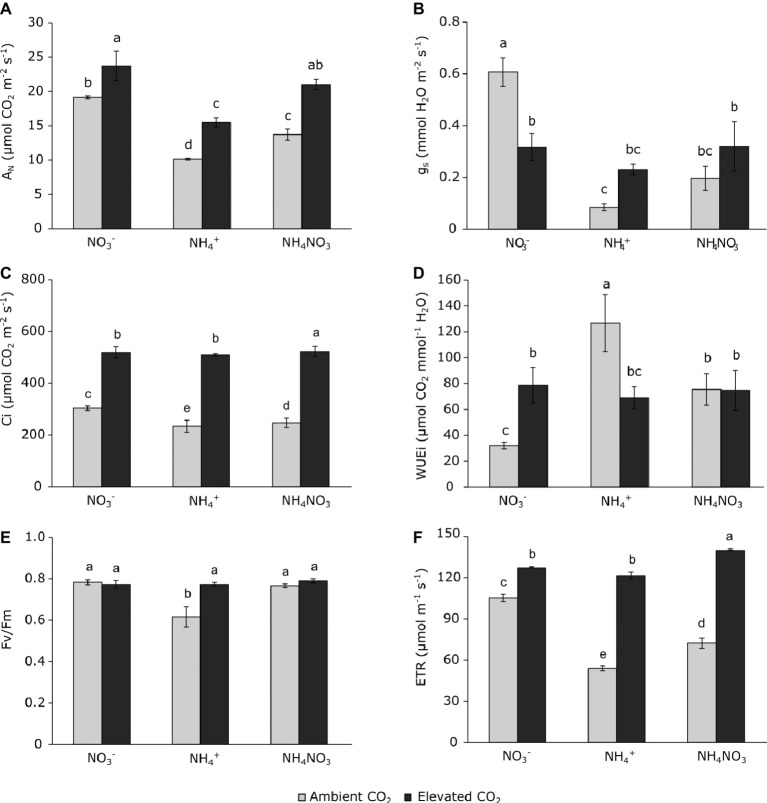
Net photosynthetic rate **(A)**, stomatal conductance **(B)**, intercellular CO_2_ mole fraction **(C)**, instantaneous water use efficiency **(D)**, maximal quantum efficiency of PSII **(E)**, and thylakoid electron transport rate **(F)** of wheat plant grown under ambient (400 ppm CO_2_, grey bars) and elevated (700 ppm CO_2_, black bars) CO_2_ conditions. Significant differences (*p* < 0.05) between N treatments and CO_2_ conditions are indicated with different letters. Values represent mean ± SEM (*n* = 3).

Gas-exchange parameters measured in flag leaves of wheat plants at Z51 (Zadoks scale) were more affected by N source than by CO_2_ concentration (Table 1). In addition, the effect size of each factor was different (Table 1). Ammonium- and ammonium nitrate-fertilized plants grown under ambient CO_2_ conditions had a lower CO_2_ assimilation rate (A_N_), stomatal conductance (g_s_), and intercellular CO_2_ concentration (C_i_) than nitrate-fertilized plants (Figures 1A–C). Moreover, the WUEi was higher in wheat plants grown at ambient CO_2_ conditions fertilized with ammonium or ammonium nitrate than under nitrate nutrition (Figure 1D). In addition, WUEi was higher in ammonium-fertilized plants than those fertilized with ammonium nitrate (Figure 1D). Under elevated CO_2_ conditions, only plants fertilized with nitrate showed lower stomatal conductance than their respective control plants grown at ambient CO_2_ (Figure 1B), but the photosynthetic rate was higher than the respective plants grown under ambient CO_2_ conditions (Figure 1A). By contrast, plants fertilized with ammonium nutrition exposed to elevated CO_2_ showed higher photosynthetic assimilation rates and stomatal conductance than the respective plants grown under ambient CO_2_ conditions (Figures 1A,B). Interestingly, ammonium nitrate-fertilized plants also showed an enhanced photosynthetic rate at elevated CO_2_ relative to ambient CO_2_ conditions, and in such a way that they showed values equal to the nitrate-fertilized plants (Figure 1A). The exposure to elevated CO_2_ increased WUEi in nitrate-fertilized plants but decreased WUEi in ammonium-fertilized plants as a consequence of the direct CO_2_ effect on stomatal conductance (Figure 1D).

**Table 1:** Significance and size effect of each factor (CO_2_ concentration and N source) and their interactions on the different variables measured.

	CO_2_ concentration		N source		CO_2_ concentration × N source	
	Sig.	partial *η*^2^	Sig.	partial *η*^2^	Sig.	partial *η*^2^
Shoot biomass	n.s.	0.138	n.s.	0.329	n.s.	0.26
A_N_	***	0.792	***	0.852	n.s.	0.134
g_s_	n.s.	0.002	***	0.733	**	0.632
Ci	***	0.979	***	0.929	***	0.915
WUEi	n.s.	0.01	*	0.444	**	0.547
*F*_v_/*F*_m_	*	0.414	**	0.577	**	0.537
ETR	***	0.982	***	0.93	***	0.916
*TIP1*	n.s.	0.147	**	0.42	n.s.	0.063
*PIP1.1*	n.s.	0.134	**	0.421	n.s.	0.266
*PIP2.3*	n.s.	0.111	n.s.	0.135	n.s.	0.133
*AMT2.1*	***	0.685	*	0.315	n.s.	0.119
*NRT1.1*	n.s.	0.186	n.s.	0.099	n.s.	0.045
*NRT1.7*	n.s.	0.205	n.s.	0.119	*	0.309
Soluble starch synthase	n.s.	0.189	n.s.	0.234	n.s.	0.276
Total amylase	***	0.509	**	0.527	n.s.	0.287
Starch	n.s.	0.201	n.s.	0.167	n.s.	0.223
Maltose	***	0.505	*	0.3	n.s.	0.057
NH_4_^+^	***	0.496	***	0.716	n.s.	0.002
Free amino acids	***	0.746	***	0.671	n.s.	0.158
Soluble protein	***	0.632	*	0.081	n.s.	0.135
Proline	***	0.85	***	0.811	n.s.	0.336
NR	*	0.21	***	0.68	n.s.	0.06
GS	***	0.502	***	0.654	*	0.377
Aminating-GDH	n.s.	0.045	n.s.	0.154	**	0.446

* p < 0.05;

** p < 0.01;

*** p < 0.001.

**Table 2:** Shoot biomass (g DW plant^−1^) of wheat plants grown under different N source and ambient (400 ppm CO_2_) or elevated CO_2_ conditions (700 ppm CO_2_).

		Shoot biomass			
400 ppm CO_2_	NO_3_^−^	21.77	±	2.73	ab
	NH_4_^+^	14.64	±	1.23	c
	NH_4_NO_3_	20.80	±	0.53	b
700 ppm CO_2_	NO_3_^−^	26.26	±	1.10	a
	NH_4_^+^	15.14	±	2.32	c
	NH_4_NO_3_	26.56	±	1.90	a

Significant differences (p < 0.05) between N treatments and CO_2_ conditions are indicated with different letters. Values represent mean ± SEM (n = 4).

*F*_v_/*F*_m_ value of 0.83 was significantly lower under ammonium nutrition at ambient CO_2_ conditions than nitrate nutrition (Figure 1E). However, both ammonium and ammonium nitrate nutrition resulted in a reduced thylakoid electron transport rate (ETR) with respect to nitrate nutrition under ambient CO_2_ (Figure 1F). At the same time, and regardless of the nitrogen nutrition, elevated CO_2_ conditions stimulated the thylakoid ETR relative to plants grown at ambient CO_2_ (Figure 1F). Although differences in ETR among the N treatments were minimal for plants grown at elevated CO_2_, the highest values being observed in ammonium nitrate-fertilized plants (Figure 1F).

**Figure 2:**
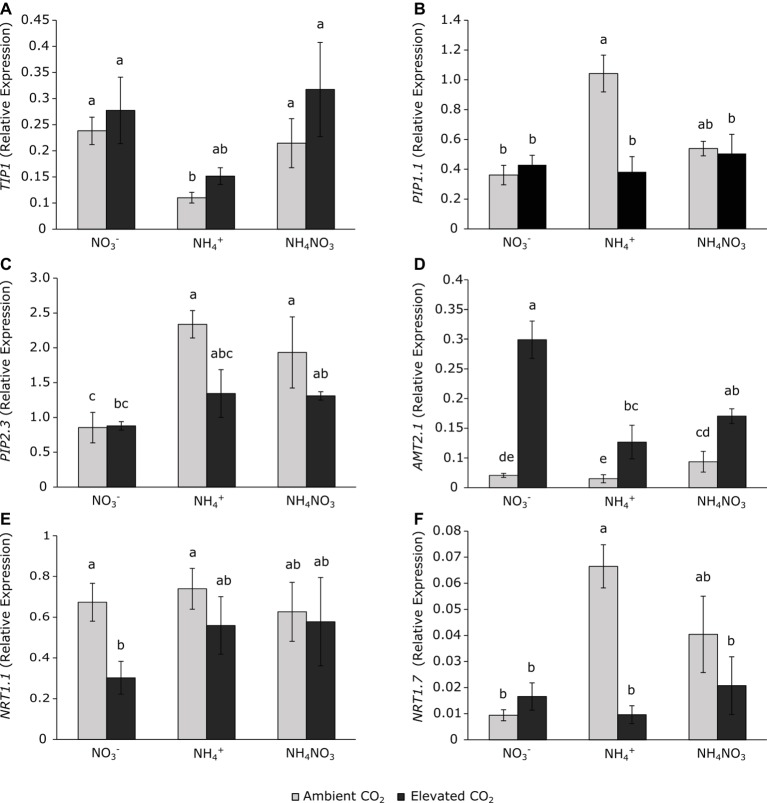
Aquaporins *TIP1* **(A)**, *PIP1.1* **(B)**, and *PIP2.3* **(C)** and on nitrogen transporters *AMT2.1* **(D)**, *NRT1.1* **(E)**, and *NRT1.7* **(F)** in leaves of wheat plants grown under ambient (400 ppm CO_2_, grey bars) and elevated (700 ppm CO_2_, black bars) CO_2_ conditions. Significant differences (*p* < 0.05) between N treatments and CO_2_ conditions are indicated with different letters. Values represent mean ± SEM (*n* = 4).

Aquaporins are protein channels involved in the flow of water, CO_2_, and even ammonia, and they can respond to environmental conditions. Aquaporin expressions were affected exclusively by the N source, but not by CO_2_ concentration (Table 1). Wheat plants grown under ammonium nutrition at ambient CO_2_ conditions presented a downregulation of tonoplast aquaporin *TIP1* (Figure 2A) with respect to nitrate-fertilized plants but had stimulated expression of the *PIP1.1* and *PIP2.3* aquaporins in the plasma membrane (Figures 2B,C). These changes in aquaporin gene expression were not detected under elevated CO_2_. Plants exposed to elevated CO_2_ conditions showed higher expression of *AMT2.1* (Figure 2D) than their respective controls at ambient CO_2_. Nitrate nutrition downregulated the expression of the *NRT1.1* (Figure 2E) nitrate transporter under elevated CO_2_ conditions, and the expression of *NRT1.7* (Figure 2F) showed a large stimulation in ammonium-fertilized plants at ambient CO_2_ conditions.

**Figure 3:**
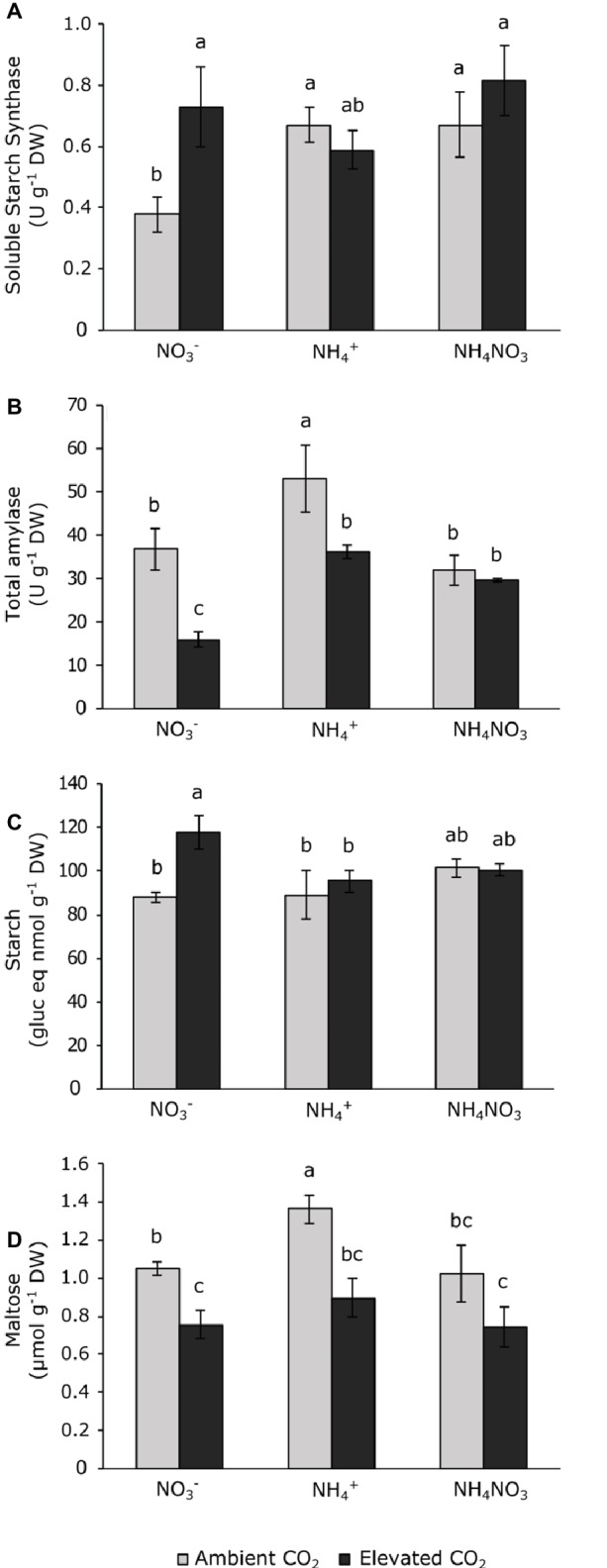
Soluble starch synthase **(A)** and total amylase **(B)** activities and carbohydrate contents of starch **(C)** and maltose **(D)** in leaves of wheat plant grown under ambient (400 ppm CO_2_, grey bars) and elevated (700 ppm CO_2_, black bars) CO_2_ conditions. Significant differences (*p* < 0.05) between N treatments and CO_2_ conditions are indicated with different letters. Values represent mean ± SEM (*n* = 4).

Photoassimilate levels were represented by the polysaccharide starch and soluble maltose, which are a product of starch degradation. Only total amylase and its product, maltose, were affected by CO_2_ concentration and N source, but not by its interaction (Table 1). Nitrate-fertilized plants exposed to elevated CO_2_ increased soluble starch synthase and reduced total amylase activity (Figures 3A,B), thus increasing the starch content relative to those grown at ambient CO_2_ (Figure 3C). Regarding maltose levels, plants fertilized with ammonium at ambient CO_2_ showed the highest contents of this sugar (Figure 3D). Furthermore, the maltose content was depleted in wheat plants, regardless the N source under elevated CO_2_ conditions.

**Figure 4:**
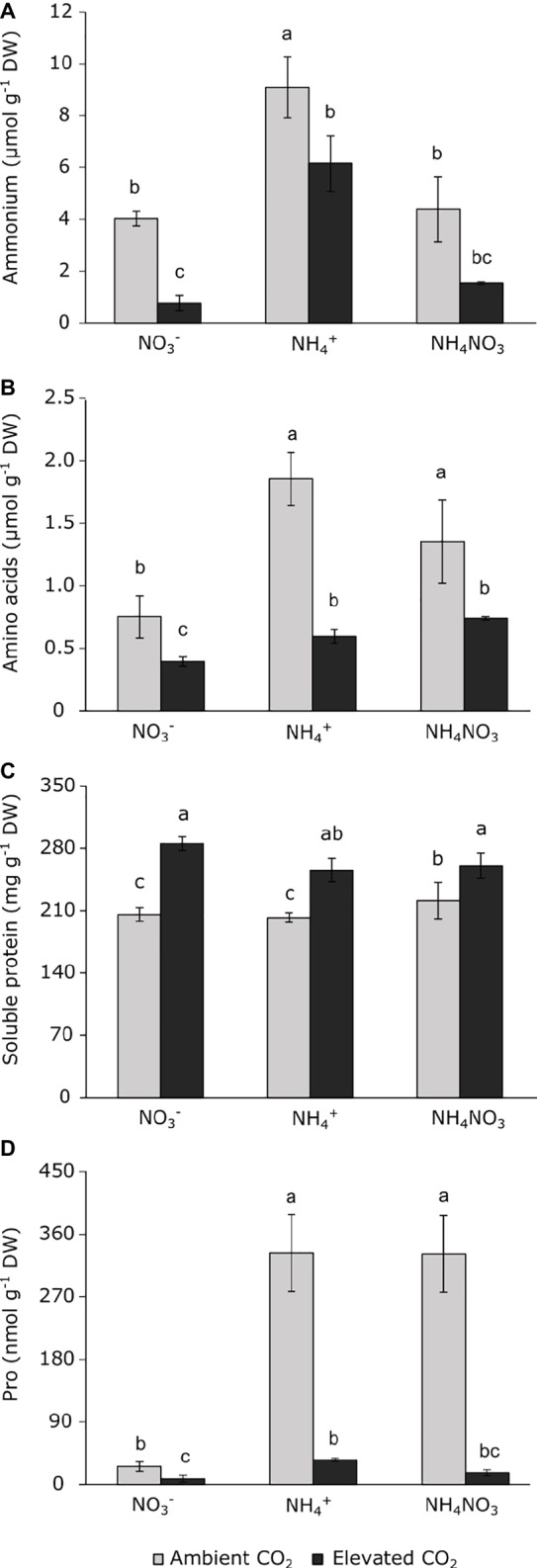
Ammonium **(A)**, amino acids **(B)**, soluble protein **(C)**, and proline **(D)** contents in leaves of wheat plants grown under ambient (400 ppm CO_2_, grey bars) and elevated (700 ppm CO_2_, black bars) CO_2_ conditions. Significant differences (*p* < 0.05) between N treatments and CO_2_ conditions are indicated with different letters. Values represent mean ± SEM (*n* = 3–4).

While no significant interaction was detected between the main factors, the two factors assayed (namely CO_2_ concentration and N source) significantly affected (Table 1) ammonium content and its incorporation in amino acids and proteins, but interactions between these factors were not observed. As expected, ammonium nutrition caused an ammonium accumulation in leaves, regardless the atmospheric CO_2_ concentration (Figure 4A). Similarly, wheat plants grown under ammonium nutrition at ambient CO_2_ conditions had the highest values of total amino acids, while exposure to elevated CO_2_ conditions reduced the total amino acid content in these plants (Figure 4B). Moreover, exposure to elevated CO_2_ increased the total soluble protein content (Figure 4C). In accordance with the amino acid contents, wheat plants grown under ammonium and ammonium nitrate nutrition showed considerably increased proline content when they were grown at ambient CO_2_. However, under elevated CO_2_ conditions, proline levels dropped drastically (Figure 4D).

**Figure 5:**
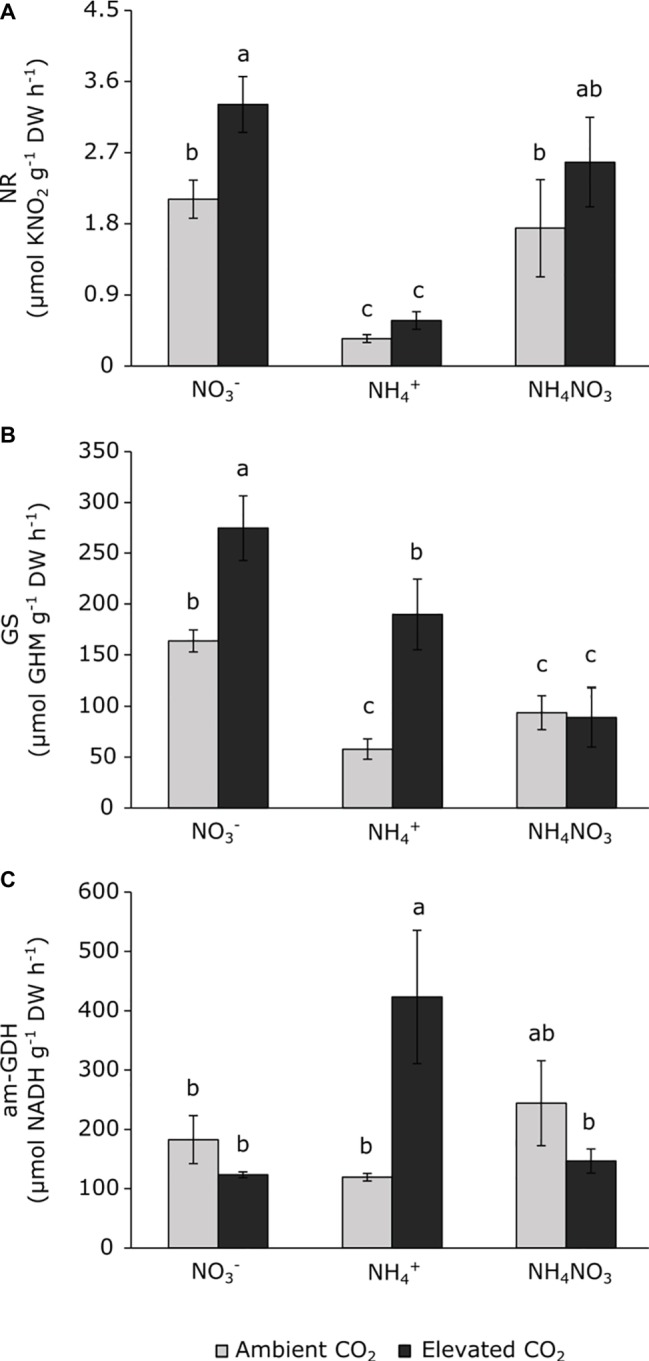
Nitrate reductase **(A)**, glutamine synthetase **(B)** and aminating glutamate dehydrogenase **(C)** activities in leaves of wheat plants under ambient (400 ppm CO_2_, grey bars) and elevated (700 ppm CO_2_, black bars) CO_2_ conditions. Significant differences (*p* < 0.05) between N treatments and CO_2_ conditions are indicated with different letters. Values represent mean ± SEM (*n* = 4).

The activity of N-metabolism enzymes changed depending on the CO_2_ levels and the type of N nutrition (Figure 5). Under ambient CO_2_ conditions, plants grown under ammonium nutrition showed lower NR and GS activities than nitrate nutrition (Figures 5A,B) and ammonium nitrate nutrition reduced the GS activity with respect to nitrate nutrition (Figure 5B). The exposure to elevated CO_2_ stimulated NR and GS activities in plants fertilized with nitrate, without changes in aminating-GDH activity (Figure 5). Meanwhile, ammonium-fertilized plants exposed to elevated CO_2_ stimulated both GS and aminating-GDH activities (Figures 5B,C). Exposure to elevated CO_2_ did not stimulate GS activity in plants fertilized with ammonium nitrate, and this activity was lower than observed in nitrate-fertilized plants (Figure 5B). Neither the NR nor the GDH activities indicated any CO_2_ effect under ammonium nitrate nutrition.

## Discussion

### Stomatal Closure as Strategy to Avoid Further Leaf Ammonium Accumulation at Ambient CO_2_ Conditions

Ammonium fertilization often causes many toxicity symptoms in plants when it is supplied as the sole N source (Britto and Kronzucker, 2002). The overall reduction in growth of wheat shoots in the present work was clear evidence of the noxious effect of ammonium when it is employed as an exclusive N source, and the overcoming of this growth limitation under mixed nutrition with ammonium nitrate lend further support to this finding (Table 2). In agreement with other studies (Setién et al., 2013; Sarasketa et al., 2016; Piñero et al., 2018), our results show that wheat plants grown under exclusive ammonium nutrition at ambient CO_2_ conditions accumulate ammonium in leaf tissues (Figure 4), reflecting the imbalance between its uptake and its assimilation into organic molecules. Indeed, it is the disruption of cell ammonium homeostasis that leads eventually to poor shoot performance (Table 2). Ammonium-fertilized plants underwent drastic stomatal closure (depletion of g_s_) at ambient CO_2_ conditions, which might be a strategy to avoid further ammonium accumulation in leaves. However, this depletion of stomatal conductance caused an inhibition of photosynthetic activity, which has important consequences for plant productivity. Therefore, ammonium fertilization had an impact on photosynthetic assimilation by limiting the CO_2_ supply to Rubisco as a result of the depletion of stomatal conductance and subsequent depletion of the intercellular CO_2_ concentration (Ci). The regulation of stomata has been described as a process linked to transport and absorption of nutrients (Aranjuelo et al., 2014). This strategy has been reported under abiotic stresses such as salinity (Wang et al., 2017) or high temperature (Aranjuelo et al., 2006; Jauregui et al., 2015), among other factors.

Water and nutrient movements through the plant and, more specifically, into the cell require a large number of processes in addition to regulation of the stomata and transpiration. Among them, aquaporins are the major factors that regulate the flow of water through the cell. In plants, the main subfamilies of aquaporins are the plasma membrane intrinsic proteins (PIPs) and tonoplast intrinsic proteins (TIPs; Forrest and Bhave, 2007). Likewise, each subfamily is subdivided into groups with various isoforms, which may be present in specific locations or have specific functions. Therefore, although the main role attributed to aquaporins is water transport, the expression of these proteins has also been reported as regulating the transport of other molecules such as ammonia (Bertl and Kaldenhoff, 2007; Forrest and Bhave, 2007; Ding et al., 2016), CO_2_ (Flexas et al., 2006; Maurel et al., 2008), boron (Durbak et al., 2014), or silicon (Ma and Yamaji, 2006; Rios et al., 2017). According to Flexas et al. (2006), the higher levels of *PIP1.1* and *PIP2.3* expression detected in ammonium-treated plants at ambient CO_2_ (Figure 2) could be a mechanism to overcome the CO_2_-supply limitations that chloroplasts experience under stomatal and mesophyll resistance. Indeed, enhanced aquaporin expression has been considered as a plant strategy for facilitating CO_2_ diffusion to carboxylation sites in order to maintain the photosynthetic rate (Ding et al., 2016). Furthermore, the regulation of aquaporin genes might be involved in regulating the uptake of water, ammonia, and other molecules in order to redistribute them throughout the cells (Forrest and Bhave, 2007). Accordingly, the expression of the *PIP1.1* and *PIP2.3* genes (Figure 2) in wheat plants fertilized under ammonium nutrition would explain the efficient use of water by the plant (WUEi) in order to coordinate ammonium accumulation, CO_2_ assimilation, and H_2_O diffusion. However, the expression of the tonoplast aquaporin *TIP1* was downregulated by ammonium under ambient CO_2_ conditions. AQPs genes are suggested to be involved in the responsiveness to osmotic stress, saline, and chilling stress (Forrest and Bhave, 2008). Therefore, the pattern of downregulation of *TIP1* (Figure 2) observed in ammonium-fertilized plants resembled that of stomatal conductance (Figure 1) and its regulation could be involved in water loss from the vacuole and stomatal closure as a way to reduce the water loss from leaves and to maintain turgidity under stress conditions (Forrest and Bhave, 2007). The regulation of tonoplast AQPs can be considered as evidence of its potential role in the regulation of water or ammonia flow through the vacuole.

On the other hand, it should be also observed that the fact that the depletion in A_N_ and g_s_ values in ammonium-fertilized plants grown under ambient CO_2_ was more marked than the diminishment in Ci reveals that Rubisco carboxylation together with altered CO_2_ diffusion within the chloroplast would strongly condition photosynthetic performance. In addition, the lower *F*_v_/*F*_m_ detected under ammonium nutrition indicated that ammonium-fertilized plants presented stress symptoms that might have affected Rubisco activity. On the other hand, both gas exchange and the AQPs gene expression analyses would also support the idea that CO_2_ diffusion from substomatal cavity toward chloroplast (referred to as mesophyll conductance; *g*_m_) would have been affected in plants fertilized with ammonium. Previous studies carried out in plants exposed to different environmental conditions, such as leaf CO_2_, temperature, and water stress remark how *g*_m_ limits photosynthesis by the availability of CO_2_ within the chloroplast and how AQPs are involved in *g*_m_ regulation (Bernacchi et al., 2002; Flexas et al., 2006, 2007; Galmés et al., 2007). Our study showed that ammonium exposure (provides as mixed or sole N source) constrained, in a first step stomatal conductance with the consequent CO_2_ diffusion limitation into the chloroplast, and in a second step net photosynthesis of plants grown at ambient CO_2_.

The depletion of the *F*_v_/*F*_m_ index indicates that photo-inhibition occurred due to ammonium exposure (Figure 1). Indeed, stress symptoms caused by an intracellular redox imbalance within the mitochondrial electron transport chain have been described when plants are grown under ammonium nutrition (Jauregui et al., 2017; Liu and Von Wirén, 2017). Thus, our results might indicate that ammonium accumulation in leaves could have contributed to the appearance of reactive oxygen species (ROS), causing a depletion in the *F*_v_/*F*_m_. Moreover, our study supports the fact that the inhibition of a major electron sink (i.e., photosynthetic CO_2_ assimilation) would be involved in damage to PSII elicits an imbalance in the electron source to CO_2_ assimilation with the consequent photo-inhibition. In agreement with this finding, the enhanced proline content in ammonium-fed leaves (Figure 3) could have played a role in the response to the stress symptoms in the cell. Proline content is widely reported to increase under certain abiotic stresses, such as temperature or osmotic stress to prevent oxidative damage (Szabados and Savouré, 2009). Proline is a glutamate-derived amino acid; however, under certain stress conditions, maltose, which is a transitory product derived from starch degradation, can be connected with proline biosynthesis (Zanella et al., 2016). In this way, the high maltose content observed when wheat is fertilized with ammonium (Figure 3) could support the biosynthesis of proline (Zanella et al., 2016; Baslam et al., 2017) targeted toward neutralizing the stress symptoms elicited by ammonium accumulation.

Transpiration is a key process for the transport and movement of water and other compounds, either essential nutrients or xenobiotics, throughout the plant body. This process is greatly affected by ammonium nutrition in wheat plants. In parallel with other compounds that impair photosynthetic machinery *via* stomatal regulation (Aranjuelo et al., 2014) or to the “avoidance mechanism” under saline stress (Wang et al., 2017), ammonium-fertilized plants are able to regulate their stomatal function and thus prevent further ammonium accumulation in leaves *via* the transpiratory stream, which otherwise could be extremely harmful to cell functions. The counterbalance for the plant is that stomatal closure restricts the entrance of CO_2_, with negative consequences for photosynthesis and biomass productivity. Although ammonium nitrate also reduced stomatal conductance and ETR, the presence of half of the nitrogen in the form of nitrate might have reduced the ammonium toxicity under ambient CO_2_ conditions.

### Elevated CO_2_ Ameliorates the Photosynthetic Process and Stomatal Conductance in Ammonium-Fertilized Plants, Avoiding Stress Symptoms

The enhancement of ammonium tolerance has been reported under a range of conditions such as high irradiance (Setién et al., 2013), high external pH in the growth medium (Sarasketa et al., 2016), increasing atmospheric CO_2_ (Rubio-Asensio et al., 2015; Vega-Mas et al., 2017), or fertilizing with a mixed ammonium nitrate nutrition (Zaghdoud et al., 2016). In our study, we have not observed any positive effect of elevated CO_2_ on biomass production under ammonium nutrition alone, unlike the observations under sole nitrate or mixed nutrition. Accordingly, the effect of elevated CO_2_ on the parameters related to photosynthetic performance and stomatal functioning was quite different, depending on the N source. Under elevated CO_2_, stomatal closure took place when nitrate was the N source, but contrary to this, the strong effect of ammonium on stomatal conductance observed under ambient conditions was offset during the exposure to an enriched CO_2_ atmosphere. Thus, wheat plants grown at elevated CO_2_, presented similar values of stomatal conductance and WUEi, regardless the N source (Figure 1).

The greater availability of carbon prevented photo-inhibition in the ammonium-stressed plants, which retained normal values of *F*_v_/*F*_m_ (Figure 1). Therefore, the exposure to elevated CO_2_ lessened the stress observed in ammonium-fertilized plants, as indicated by the higher electron transport due to stimulation of CO_2_ assimilation. Further, these conditions meant that the proline content was lower (Figure 4). The compensatory CO_2_ effect on these physiological processes under ammonium nutrition matches the absence of significant differences in the expression of *PIP1.1*, *PIP2.3,* and N transporters (Figure 2). This indicates that in the absence of a metabolic stress context, ammonium-fertilized plants can maintain their stomatal conductance, and this removes the necessity to adjust cell physiology toward the diffusion of CO_2_ and H_2_O through the cell membranes.

The stimulation of net photosynthetic assimilation under elevated CO_2_ would allow more carbon skeletons to be diverted to the Krebs cycle (Setién et al., 2013) to maintain ammonium assimilation. The commonly described incorporation of ammonium into amino acids *via* GS and GOGAT activities, which are enhanced under elevated CO_2_, would improve glutamine and glutamate supplies, respectively. In addition, an alternative pathway proposed to reduce ammonium excess is GDH amination, which catalyzes the conversion of α-ketoglutarate into glutamate (Setién et al., 2013; Vega-Mas et al., 2015). The disappearance of stress observed at elevated CO_2_ conditions is concordant with a greater capacity of the plant to assimilate ammonium, improving its assimilation rates and leading to C and N being accumulated as proteins rather than carbohydrates or free amino acids (Figures 4, 5).

On the other hand, elevated CO_2_ favored behavior in ammonium nitrate-grown plants that resemble ammonium-fertilized plants in terms of stomatal physiology, without any changes in WUE or AQPs and N-transporter gene expression (Figures 1, 2). Indeed, the recovery of photosynthesis was even better than under the exclusive ammonium source, which contributed toward the biomass increasing under elevated CO_2_ conditions (Table 2). Our results are in line with many reports of maximal growth rates in plants being achieved with mixed nutrition (Piñero et al., 2018; Shang and Shen, 2018).

Under different types of abiotic stress or nutrient stress, the root system behaves not only as an organ that captures resources for the plant (Ye et al., 2018), but it also serves as a storage organ or barrier that protects the aerial parts from toxic elements (Setién et al., 2014; Rios et al., 2017; Vega-Mas et al., 2017). Preferential investment of resources and carbon allocation in the root system as the first organ to encounter ammonium nutrition could explain why wheat shoot physiology and metabolism recover partially from stress under high CO_2_ availability, but that ultimately, this better performance is not translated into shoot growth.

## Conclusions

The present work underscores the impact of the N source and elevated CO_2_ on stomatal opening and plant physiology. Under ambient CO_2_ conditions, ammonium nutrition leads to toxic effects with stress symptoms and reductions in stomatal conductance and photosynthetic performance, with a consequent impact on plant biomass. Within this context, stomatal closure is interpreted as a strategy to diminish ammonium transport toward leaves due to a reduction in leaf transpiration (as also reflected in the lower expression of the *TIP1* water transporter and the *AMT2.1* ammonium transporter). Further, the gene expression analyses also highlighted that in ammonium fertilized plants, CO_2_ diffusion toward the chloroplasts might have been favored by overexpression of *PIP1.1* gene in order to overcome the impact of Ci caused by a depletion of stomatal conductance. On the other hand, the study showed that under elevated CO_2_ conditions, in which photo-inhibition symptoms disappeared, physiological parameters such as stomatal conductance together with the expression of water, ammonium, and CO_2_ transporters (*TIP1*, *AMT2.1* and *PIP1.1*, respectively) were maintained at control values. In addition, our study also revealed that in the context of rising CO_2_, ammonium-based fertilization might favor the photosynthetic performance toward greater redistribution of carbon assimilated *via* the Krebs cycle to maintain ammonium assimilation. Finally, our study also showed that the mixed ammonium nitrate nutrition could improve water use efficiency and plant productivity.

## Author Contributions

FT, IA, and CG-M designed the experiments and developed the structure. FT grew the plants, undertook the measurements, harvested the plants, and analyzed the data. EB-F analyzed the sugar content and starch synthesis and degradation activities. IA, MG-M, and CG-M supervised the experiments. FT, IA, and MG-M structured the paper, made critical revisions, and had primary responsibility for the final content. CG-M provided valuable discussion and finalized writing of the manuscript. All authors have read and approved the final manuscript.

### Conflict of Interest Statement

The authors declare that the research was conducted in the absence of any commercial or financial relationships that could be construed as a potential conflict of interest.

## Footnotes

^1^http://www.fao.org
